# Evolution of *Salmonella*-Host Cell Interactions through a Dynamic Bacterial Genome

**DOI:** 10.3389/fcimb.2017.00428

**Published:** 2017-09-29

**Authors:** Bushra Ilyas, Caressa N. Tsai, Brian K. Coombes

**Affiliations:** ^1^Department of Biochemistry and Biomedical Sciences, McMaster University, Hamilton, ON, Canada; ^2^Michael DeGroote Institute for Infectious Disease Research, McMaster University, Hamilton, ON, Canada

**Keywords:** *Salmonella* infection biology, bacterial pathogenesis, horizontal gene transfer, regulatory evolution, virulence regulation, gene loss, comparative genomics, xenogeneic silencing

## Abstract

*Salmonella* Typhimurium has a broad arsenal of genes that are tightly regulated and coordinated to facilitate adaptation to the various host environments it colonizes. The genome of *Salmonella* Typhimurium has undergone multiple gene acquisition events and has accrued changes in non-coding DNA that have undergone selection by regulatory evolution. Together, at least 17 horizontally acquired pathogenicity islands (SPIs), prophage-associated genes, and changes in core genome regulation contribute to the virulence program of *Salmonella*. Here, we review the latest understanding of these elements and their contributions to pathogenesis, emphasizing the regulatory circuitry that controls niche-specific gene expression. In addition to an overview of the importance of SPI-1 and SPI-2 to host invasion and colonization, we describe the recently characterized contributions of other SPIs, including the antibacterial activity of SPI-6 and adhesion and invasion mediated by SPI-4. We further discuss how these fitness traits have been integrated into the regulatory circuitry of the bacterial cell through *cis*-regulatory evolution and by a careful balance of silencing and counter-silencing by regulatory proteins. Detailed understanding of regulatory evolution within *Salmonella* is uncovering novel aspects of infection biology that relate to host-pathogen interactions and evasion of host immunity.

## Introduction

*Salmonella* is a genus of enteric pathogens that consists of two species, *Salmonella enterica* and *Salmonella bongori*, which can cause disease in a broad range of hosts. *S. bongori* is predominantly associated with infection in reptiles, although it has been isolated in human infections (Nastasi et al., 1988; Giammanco et al., 2002). *S. enterica* is further divided into seven subspecies, which can cause gastroenteritis or systemic disease in a variety of warm- and cold-blooded animals (McQuiston et al., 2008). Among the *S. enterica* subspecies, *S. enterica* subsp. *enterica* is the only one that can infect mammals, and is associated with human disease. This subspecies includes host-restricted serovars like *Salmonella* Typhi, which causes typhoid fever in humans, and the broad host-range *Salmonella* Typhimurium, which causes gastroenteritis in humans and other mammals (Uzzau et al., 2000). The evolution of *Salmonella* as a pathogen has been broadly studied over the past few decades. The recent rise in comparative genomics methods has cast a light on the molecular basis of pathogenesis, revealing that the evolution of *Salmonella* toward pathogenicity was mediated by several horizontal transfer events (Bäumler, 1997; Groisman and Ochman, 1997).

Genes acquired by horizontal transfer can confer new phenotypes to the recipient bacteria and are often the source of adaptive changes that maximize fitness in a given niche (Ochman et al., 2000). In the *Salmonella* genomics vernacular, horizontally acquired multi-gene loci linked to infection are called *Salmonella* Pathogenicity Islands (SPIs), and have been described as the “molecular toolbox” for *Salmonella* pathogenesis (Gal-Mor and Finlay, 2006). The SPI pan-genome includes 21 SPIs, most of which are present in both species, and across all subspecies (McClelland et al., 2001). However, there is genetic flux with the *Salmonella* species and subspecies, which define host range and disease phenotype (Bäumler, 1997; Fookes et al., 2011).

Perhaps the best-studied serovar of *Salmonella* is *S. enterica* subsp. *enterica* sv. Typhimurium (*S*. Typhimurium). *S*. Typhimurium is amenable to molecular manipulation, can infect numerous cell types, and robust animal models have been developed to model both self-limiting gastroenteritis and systemic infection (Finlay and Brumell, 2000; Tsolis et al., 2011). Together, these tools have laid the foundation for understanding the genetic basis for *Salmonella* virulence, and have helped understand the host response to infection. The contributions of SPI-1 and 2 to *Salmonella* pathogenicity have been reviewed in detail several times (Hensel, 2000; Wallis and Galyov, 2000; Waterman and Holden, 2003; Haraga et al., 2008), and more recent attention has been directed toward the evolution and virulence determinants of the other SPIs (Morgan et al., 2004; Haneda et al., 2009; Nieto et al., 2016). In this review, we highlight the contribution of horizontally acquired genes to the adaptation of *Salmonella* as a pathogen and its biology within host cells. Further, because many bacteria share a conserved repertoire of core genes, we discuss a notion that uncovering selective changes to gene deployment via regulatory evolution among conserved genes is a strategy to uncover novel aspects of infection biology.

## Distinct virulence factors drive specific stages of *Salmonella* infection

**Figure 1:**
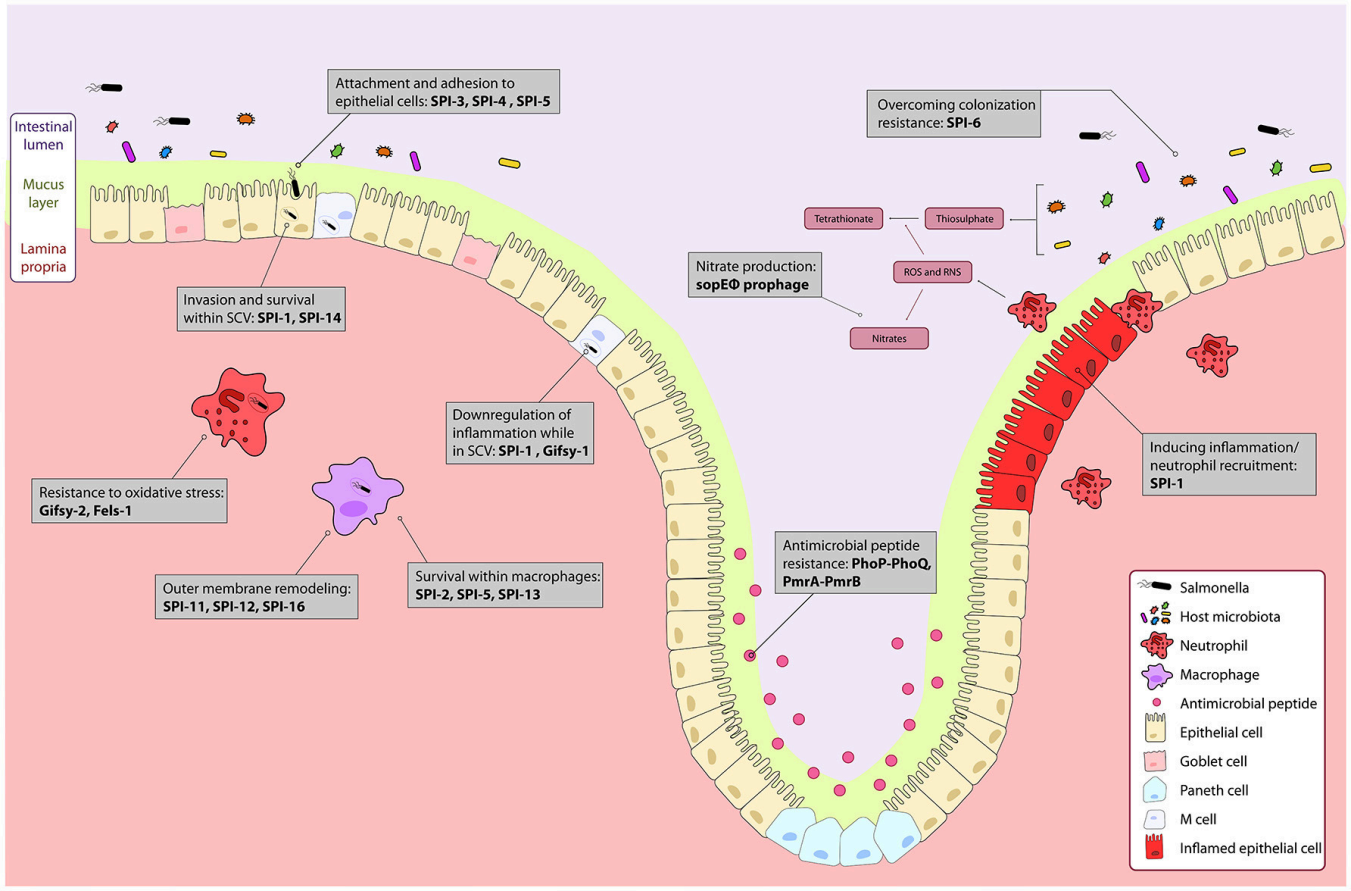
The course of *Salmonella* infection within the intestine is driven by horizontally acquired virulence factors. Genes encoded on the horizontally acquired *Salmonella* Pathogenicity Islands (SPIs) and prophage associated genes are important during *Salmonella* infection of the intestine. *Salmonella* colonizes the intestinal lumen through activation of robust inflammation by genes encoded on the sopE prophage, and SPI-1 secreted effectors, and outcompetes commensal microbes metabolically, and with a type 6 secretion system encoded on SPI-6. The PhoPQ and PmrAB two-component systems regulate resistance to luminal antimicrobial peptides. Surface proteins encoded on SPI-3 and SPI-4 drive attachment to epithelial cells, and bacterial mediated endocytosis into epithelial cells is induced by gene products from SPI-1 and SPI-5. Modification of the bacterial endosome and survival within the *Salmonella* containing vacuole (SCV) is driven by SPI-1 and the Gifsy-1 prophage. Within the SCV and in immune cells like neutrophils and macrophages, resistance to immune responses such as oxidative stress is mediated by the Gifsy-2 and Fels-1 prophages, and SPI-11, SPI-12, and SPI-16 that remodel the outer membrane to evade immune responses. Survival within macrophages is mediated by SPI-2, SPI-5, SPI-13. Together, these genes allow for colonization and systemic infection of *Salmonella* Typhimurium.

Genetically susceptible mice (*Nramp/SLC11A1*^−/−^) orally infected with *S*. Typhimurium develop systemic disease, characterized by high bacterial burdens in the spleen and liver, gross intestinal pathology, and death from systemic bacteremia (Santos et al., 2001; Cuellar-Mata et al., 2002). Streptomycin pre-treatment in this model lowers intrinsic colonization resistance and intensifies the bacterial-driven intestinal inflammation, a finding that has been extensively leveraged to understand how *Salmonella* competes metabolically in the inflamed intestine (Barthel et al., 2003; Winter et al., 2010a,b). During the infection process, *S*. Typhimurium invades epithelial cells or is taken up by M cells, colonizes the Peyer's patches and associated lymphoid tissue, and invades or is phagocytosed by immune cells such as macrophages and neutrophils. In this permissive niche for replication, systemic dissemination proceeds to shuttle salmonellae to sites such as the spleen and liver. At each step in this process, specific virulence factors (summarized in Table 1) are activated to interact with the host to make the environment more conducive to bacterial survival and replication. Below, we describe the three major stages of *Salmonella* infection and the genetic basis for pathogenesis at each one (Figure 1).

**Table 1:** Summary of horizontally acquired genes and their role in *Salmonella* interaction with host cells.

**Virulence factor**	**Gene location**	**Activity**	**Role in infection**
SipA, SipB	SPI-1	Actin binding	Membrane ruffling, invasion (Raffatellu et al., 2005)
SseF, SseG	SPI-2	Interacts with microtubules	SCV localization, intracellular survival (Hensel et al., 1998)
MisL	SPI-3	Binding fibronectin	Attachment, long term colonization (Dorsey et al., 2005)
SiiE	SPI-4	Adhesion to epithelial cells	Attachment to intestinal epithelium, aids in invasion (Morgan et al., 2007)
SopB	SPI-5	Interaction with Rho-GTPase	Membrane ruffling, invasion of epithelial cells, activation of pro-inflammatory response (Perrett and Zhou, 2013)
Tae4	SPI-6	Anti-bacterial type 6	Overcoming colonization resistance, luminal colonization (Sana et al., 2016)
SciG, SciS	SPI-6	Unknown	Intracellular survival, systemic infection (Mulder et al., 2012)
PagC, PagD, EnvE, EnvF	SPI-11	*Salmonella* outer membrane remodeling	Resistance to antimicrobial peptides, survival within macrophages (Miller et al., 1989; Gunn et al., 1995)
SspH2	SPI-12	Interaction with NLR's	Immune evasion, intracellular survival (McGhie et al., 2009)
STM3118, STM3119	SPI-13	Peptidoglycan remodeling, putative monoamine oxidase	Immune evasion, survival within macrophages (Haneda et al., 2009)
LoiA	SPI-14	Regulation of *hilD* on SPI-1	Regulation of SPI-1 genes, important for invasion (Jiang et al., 2017)
STM0557	SPI-16	O-antigen modification	Serotype conversion, long term colonization (Bogomolnaya et al., 2008)
SopE	SopEΦ prophage, present in subset of *S*. Typhimurium isolates	Interaction with caspase-1	iNOS activation, luminal colonization (Mirold et al., 2001; Lopez et al., 2012)
GogB	Gifsy-1	Interaction with ubiquitin ligase	Inhibition of pro-inflammatory response, survival within macrophages (Pilar et al., 2012)
SodC	Gifsy-2 phage, Fels-1 phage	Superoxide dismutase	Resistance to oxidative stress, survival within SCV (Ehrbar and Hardt, 2005)
SspH1	Gifsy-3 phage, present in subset of *S*. Typhimurium isolates	Ubiquitin protein ligase	Immune suppression, survival within SCV (Ehrbar and Hardt, 2005; Haraga and Miller, 2006)

### Luminal colonization

Following oral inoculation of mice, *Salmonella* face the challenge of colonizing the intestinal lumen prior to invasion and dissemination to systemic sites. An uncontrived gut microbiota limits nutrient availability and provides colonization resistance toward this first infection step (Patel and McCormick, 2014; Erhardt and Dersch, 2015). Considerable research has investigated how *Salmonella* overcomes such host restrictions within the intestinal lumen, revealing a key role for horizontally acquired genes in endowing *Salmonella* with the ability to outcompete commensal microbes to establish an intestinal niche. A recent study identified direct killing of commensal microbes by *Salmonella* through the expression of a type 6 secretion system (T6SS), a widely-distributed nano-machine used primarily for inter-bacterial antagonism (Sana et al., 2016). The Salmonella T6SS, encoded on the horizontally acquired island SPI-6—formerly Salmonella centisome 7 genomic island (SCI 7; Blondel et al., 2009)—encodes an antibacterial amidase (Tae4) that can induce bacterial lysis in non-immune target cells (Sana et al., 2016). This ability seems to confer a fitness benefit to Salmonella at later stages of colonization of the gut lumen, but not systemic sites of infection (Sana et al., 2016, 2017). Interestingly, accessory genes within the SPI-6 island also contribute to intracellular replication and systemic dissemination in the permissive mouse line, C57BL/6 (Mulder et al., 2012). These data suggest that in addition to a role in antibacterial antagonism in the gut, the Salmonella T6SS appears to also have a secondary role in an unknown facet of intracellular cell biology. In other bacteria, the T6SS is involved in dampening host immunity (Chen et al., 2017), promoting intracellular growth in macrophages (Eshraghi et al., 2016), and interacting with the host microtubule network (Sana et al., 2015), all of which point to an expanding role for T6SS in host-pathogen interactions.

Induction of inflammation is another host defense mounted in response to *Salmonella* in the lumen. Surface proteins on *Salmonella*, like flagellin and LPS, are detected by pattern recognition receptors (PRRs) on epithelial and immune cells to activate innate immune responses (Gewirtz et al., 2001). This pro-inflammatory environment is associated with a rapid neutrophil influx, leading to induction of oxidative and nitrosative stress that targets both pathogenic and commensal bacteria in the lumen (Tükel et al., 2006). *Salmonella* actively induces intestinal inflammation, and this inflammatory response is important for successful luminal colonization (Stecher et al., 2007). One of the key mediators of this intestinal inflammation are the gene products of the *Salmonella* pathogenicity island 1 (SPI-1; Lee et al., 2000). This island encodes a type 3 secretion system (T3SS-1) that translocates effector proteins into host cells to modulate their function. Effector proteins encoded within SPI-1 and in other horizontally acquired islands are translocated through the T3SS-1, and can induce inflammation within the gut. The interaction of the SPI-1 translocated proteins SopB, SopE, and SopE2 with epithelial cell Rho GTPases activates NF-kB and eventually leads to the release of pro-inflammatory cytokines such as IL-1β and IL-23 (Santos et al., 2009). Certain SPI-1 secreted proteins, such as SipC, SipD, and SopE have been shown to induce iNOS expression in macrophages, and recent research has demonstrated that this induction, specifically by SopE, promoted *Salmonella* colonization (Cherayil et al., 2000; Lopez et al., 2012). The mechanistic basis for the enhanced growth of *Salmonella* in the inflamed gut appears to be largely metabolic fitness. Much work has demonstrated that *Salmonella* thrives in inflamed environments because it can better utilize metabolic by-products generated during inflammation. For example, nitrates produced from reactive oxygen and nitrogen products within the lumen, and tetrathionate produced from reduced thiosulfate, provide alternative energy sources for *Salmonella*, which can be metabolized in the anaerobic gut environment (Winter et al., 2010b; Lopez et al., 2012). *Salmonella*-specific genes such as the tetrathionate utilization operon (*ttr*) allow the bacteria to utilize these alternate metabolites to outcompete commensal microbes (Hensel et al., 1999; Rivera-Chávez et al., 2013). Although nitrate respiration is conserved among *Proteobacteria*, the existence of a secretion system effector that manipulates the host in order to preferentially generate nitrates has only been reported in *Salmonella* (Lopez et al., 2012). In addition to the metabolic benefits of oxidative and nitrosative stress, *Salmonella* encodes redundant systems of defense against these reactive oxygen and nitrogen intermediates, acting to mitigate damage under high concentrations (Vazquez-Torres et al., 2000; Aussel et al., 2011; Henard and Vázquez-Torres, 2011).

Within the lumen, salmonellae also encounter antimicrobial peptides secreted by epithelial cells and infiltrating immune cells, which work to disrupt membrane integrity (Bevins and Salzman, 2011). *Salmonella* has a number of genes that modify the outer membrane to protect against this host defense activity. Many of these genes are regulated by the PhoPQ and PmrAB two-component systems (Gunn and Miller, 1996). Horizontally acquired genes have also been implicated in the defense to antimicrobial peptides. For example, *Salmonella* can inhibit antimicrobial peptide production by Paneth cells in a T3SS-1-dependent manner (Salzman et al., 2003). This is likely mediated through the modification of host cell signaling pathways induced by SPI-1 effector proteins.

### Invasion of epithelial cells is a cooperative multi-gene process

The attachment and invasion of *Salmonella* to host epithelial cells sets up conditions that eventually favor luminal colonization and systemic infection. Epithelial cells in the gut, specifically goblet cells, produce mucins that form a physical barrier between the apical surface of these cells and the lumen (McGuckin et al., 2011). Zarepour et al. showed that this mucus barrier is an important host defense against *Salmonella* (Zarepour et al., 2013). Virulent *Salmonella* strains can bind to mucin preparations better than avirulent strains, suggesting the genetic factors involved in *Salmonella* virulence mediate this binding, although the interacting partners have not been identified (Vimal et al., 2000). As *Salmonella* approaches the epithelial barrier within the intestine, it can be endocytosed by M cells (Clark et al., 1994). Entry into the lamina propria allows for TLR activation by flagellin on the basolateral surface, uptake by macrophages, and activation of B and T cells (Johansson et al., 2006). In addition to luminal sampling by M cells, *Salmonella* can mediate uptake by host epithelial cells through a cooperative interaction among several virulence factors. Genes on SPI-4, which encode a Type 1 secretion system and major adhesin protein SiiE, are important for initiating epithelial cell contact in the intestine (Gerlach et al., 2007). The activity of SiiE is important for maximal bacterial entry into polarized epithelial cells because it allows for clusters of bacteria in close proximity to the invasion site to be pulled into the membrane ruffle at the host cell surface (Lorkowski et al., 2014). In addition to SiiE in SPI-4, the horizontally acquired gene *misL*, encoded on SPI-3, binds fibronectin and is required for long-term colonization (Dorsey et al., 2005). Along with the protein ShdA, which is not encoded on a genomic island, binding to surface structures like fibronectin appears to be important for intestinal persistence (Kolachala et al., 2007). The activation of intestinal inflammation can increase levels of fibronectin on epithelial cells, and MisL has been suggested to bind fibronectin at areas of epithelial cell erosion, thus providing another example of the link between intestinal inflammation and efficient bacterial invasion.

The T3SS-1-mediated invasion of *Salmonella* has been extremely well-studied and the specific role of SPI-1 associated effectors in this process have been reviewed elsewhere (Lostroh and Lee, 2001; McGhie et al., 2009; Que et al., 2013). The net result of effector translocation upon host cell contact is the induction of major cytoskeletal changes in the epithelial cell, leading to membrane ruffling and bacterial internalization. The effectors involved in this process are encoded in SPI-1 and elsewhere in the genome and are coordinated at the level of gene regulation for synchronized expression. Novel functions and regulatory interactions are still being uncovered for even well-known SPI-1 effectors. For example, the phage-encoded effector, SopE, can exert an inside-out response on host epithelial cells by enhancing their expression of iNOS, which in turn liberates host-derived nitrate that luminal *Salmonella* can respire anaerobically (Lopez et al., 2012). The SPI-5 genomic island is another mosaic island that enables different stages of the *Salmonella* infection process. The SPI-5 effector, SopB, is expressed and translocated by the T3SS-1 early in infection to promote membrane ruffling and invasion, whereas PipB (also encoded in SPI-5), is activated when *Salmonella* is within host cells and is translocated by the SPI-2-encoded type 3 secretion system (T3SS-2) to promote intra-macrophage survival (Knodler et al., 2002). Recently, SPI-14 was identified as being involved in *Salmonella* virulence by mediation of bacterial invasion, however the gene responsible appears to play a role in the activation of SPI-1 genes, rather than directly interacting with host processes (Jiang et al., 2017).

Shortly after bacterial invasion, effectors secreted by SPI-1 control reversion of the host epithelial cell to its normal state, inducing de-ruffling of the membrane and repression of the pro-inflammatory signals. This is typically mediated by host ubiquitination pathways that target SPI-1 effector proteins for degradation. However, there are also specific effectors secreted through SPI-1 that modify the *Salmonella* containing vacuole (SCV). For example, the phage-encoded effector SspH2, translocated by SPI-1, helps to down-regulate pro-inflammatory responses once *Salmonella* is in the SCV (McGhie et al., 2009). SPI-1 effectors can recruit host factors to the vacuolar membrane to redirect the vacuole in the endosomal trafficking pathway, thus limiting lysosomal fusion. SPI-1 associated genes are also implicated in escape from the SCV in non-phagocytic cells, allowing for *Salmonella* survival and replication in the cytosolic compartment. This has been recently characterized in a number of epithelial cell lines (Knodler et al., 2010, 2014). The hyper-replication seen in the cytosol is associated with *Salmonella* induced pyroptosis of host cells, releasing primed bacteria into the lumen where they can infect other cells, or be picked up by macrophages. A subset of SCV's are subject to destabilization by SPI-1 associated genes, leading to a dual lifestyle of *Salmonella* within epithelial cells (Knodler, 2015).

### *Salmonella* survival within host immune cells

Whether through bacterial mediated pyroptosis, trafficking of the SCV to the basolateral surface of epithelial cells, or by direct sampling of luminal bacteria by dendritic cells, *Salmonella* eventually arrives in the lamina propria where it is taken up by phagocytic cells through a combination of bacterial-mediated endocytosis and immune cell driven phagocytosis (Haraga et al., 2008). Within immune cells, several horizontally acquired virulence determinants are deployed in the creation of the SCV and the evasion of host immune defenses. The virulence genes necessary for bacterial survival within macrophages are predominantly located on the SPI-2 genomic island that encodes the T3SS-2 and two translocated effectors, SseF and SseG (Ochman et al., 1996; Shea et al., 1996). We will not review these genes in detail as they have been comprehensively reviewed elsewhere (Cirillo et al., 1998; Hensel et al., 1998; Haraga et al., 2008; Figueira and Holden, 2012). However, a number of phage associated genes and genes encoded on other genomic islands are paramount to the survival of *Salmonella* within macrophages. For example, SPI-12 encodes *sspH2*, which is secreted by the T3SS-2. SPI-12 is a pathogenicity island relevant for immune evasion that was first linked to virulence in a calf model of *S*. Typhimurium infection (Hansen-Wester and Hensel, 2001, 2002). Transcription of the *sspH2* gene is induced in an SsrB-dependent manner during survival within RAW264.7 macrophages, and *sspH2* mutants do not induce lethal infection of calves (Miao et al., 1999; Tomljenovic-Berube et al., 2013). Also present within SPI-12 is *oafA*, which acetylates O-antigen on its 2-hydroxyl group to generate the O5 serotype, which confers antigenic variation beneficial for pathogenesis and immune evasion (Slauch et al., 1996). Similarly, SPI-5 encodes *pipA*, another effector translocated by SPI-2 that if deleted, reduces virulence in a mouse infection (Knodler et al., 2002).

The importance of bacteriophages to the spread of virulence determinants through horizontal gene transfer is well-established (Barksdale and Arden, 1974; Cheetham and Katz, 1995; Waldor, 1998). In line with this, several pathogenicity islands in the *Salmonella* genome are proximal to either phage-encoding genes or phage attachment sites (Blanc-Potard and Groisman, 1997; Wood et al., 1998). Of pathogenic importance are two prophage-like genomic regions harbored within *S*. Typhimurium, *Gifsy-1* and *2*. Both of these genetic elements influence the interactions of *Salmonella* with host cells, as was identified due to their transcriptional induction upon exposure to reactive oxygen species. Notably, *Gifsy-2* encodes the *sodC* periplasmic superoxide dismutase that confers resistance to oxidative stress (Figueroa-Bossi and Bossi, 1999). Variants of *S*. Typhimurium that are cured of these prophage become rapidly re-lysogenized upon exposure to hydrogen peroxide, indicating the importance of these genetic elements for pathogenesis and survival *in vivo* (Figueroa-Bossi and Bossi, 1999; Figueroa-Bossi et al., 2001). The *Gifsy-1* prophage encodes *gogB*, an effector that can be translocated by both SPI-1 and 2 (Coombes et al., 2005a). GogB targets the host SCF E3 type ubiquitin ligase through an interaction with Skp1 and the human F-box only 22 (FBXO22) protein to inhibit the pro-inflammatory response once *Salmonella* is in the intracellular niche (Pilar et al., 2012). Another phage relevant for infection is *Fels-1*, which carries a third *sodC* gene, again contributing to the resistance response to oxidative stress (Brüssow et al., 2004).

Within the macrophage environment, both sequestration of essential metals as well the fusion of granules containing antimicrobial peptides to the SCV act together to restrict bacterial replication. SPI-11 encodes several genes that are critical for pathogenesis and survival at this stage of infection (Miller et al., 1989). This pathogenicity island is present in both the Typhimurium and Typhi serovars, however it has undergone degradation to some extent within Typhimurium. Of particular importance are a set of genes that encode the envelope proteins, *pagD, envE, envF* (Gunn et al., 1995). These genes participate in outer membrane remodeling that improves resistance to the immune defenses encountered intracellularly. Additional important SPI-11 genes are *pagC* and *pagD*, which are part of the PhoPQ regulon and were likely acquired via horizontal gene transfer (Gunn et al., 1995). Transposon insertions within these genes have been shown to attenuate virulence and restrict survival within macrophages (Miller et al., 1989).

The systemic spread of *Salmonella* to the liver and spleen is contingent upon survival within the macrophage environment; mutants in *Salmonella* that cannot survive within this environment are avirulent in a systemic mouse model of infection. *Salmonella*-containing macrophages enter the mesenteric lymph node, where they are shuttled to the liver and spleen (Watson and Holden, 2010). Replication within macrophages is also essential for colonization at the liver and spleen, as analyses of cell types within these tissues containing *Salmonella* identify macrophages, and to a degree neutrophils, as the primary cells at infection foci (Geddes et al., 2007; Thöne et al., 2007). Genes encoded on SPI-13 are important for intracellular viability (Shi et al., 2006). SPI-13 contains a cluster that encodes putative lyase, hydrolase, oxidase, arylsulphatase regulators, and deletion of this island renders *Salmonella* attenuated for virulence (Haneda et al., 2009). STM3118 (a homolog of acetyl-coA hydrolase) is an important virulence factor contained within this island that hydrolyzes acetyl-coA to acetate in order to modify peptidoglycan as a protective mechanism against degradative enzymes found in macrophages. Additionally encoded in SPI-13 is STM3119, a monoamine oxidase that converts aminoacetone (degradation product of L-threonine) to a peptidoglycan precursor (Shi et al., 2006). Further characterizing the function of genes encoded on SPI-13 is likely to broaden our understanding of how *Salmonella* modulates the intracellular environment and interacts with the host during systemic infection.

SPI-16 is a pathogenicity island that contributes to immune evasion in the form of O-antigen variation. It includes the gene *STM0557* that resembles bactoprenol-linked glucose translocases (for example *gtrA/B*), and is predicted to be involved in serotype conversion through glycosylation of O-antigen. The *gtr* cluster of genes encodes proteins that add glucose residues to repeating O-antigen subunits within LPS, and ultimately confer form variation at the O-12 antigen galactose to generate the 12-2 variant. Most commonly, this variant arises after exposure to or growth within macrophages (Bogomolnaya et al., 2008). It has been demonstrated that form variation within O-antigen is critical for persistence of *Salmonella* infection in the murine intestine, and so SPI-16 may play a role in the re-infection of the intestine from the gall bladder once *Salmonella* has established a niche in the liver in a chronic model of *Salmonella* infection.

Taken together, the suite of horizontally acquired virulence genes found across the SPIs act in a concerted fashion to drive infection, as *Salmonella* successfully outcompetes the host microbiota, colonizes the lumen, invades the intestinal epithelium, and resides within the replicative niches of neutrophils and macrophages. At various stages of the pathogenesis process, *Salmonella* encounters several host defense mechanisms deployed by the immune system. However, it appears that *Salmonella* has evolved to integrate these immune response cues into signaling pathways leading to adaptive gene expression that, ultimately, evades these very host defenses (Wong et al., 2009; Arpaia et al., 2011). In severe stages of systemic infection, the establishment of infection foci in the liver and spleen eventually leads to necrotic lesions and death of susceptible mice through lipid A induced toxic shock (García-Del Portillo, 2001; Santos et al., 2001).

## Regulatory evolution and transcriptional rewiring in *Salmonella*

**Figure 2:**
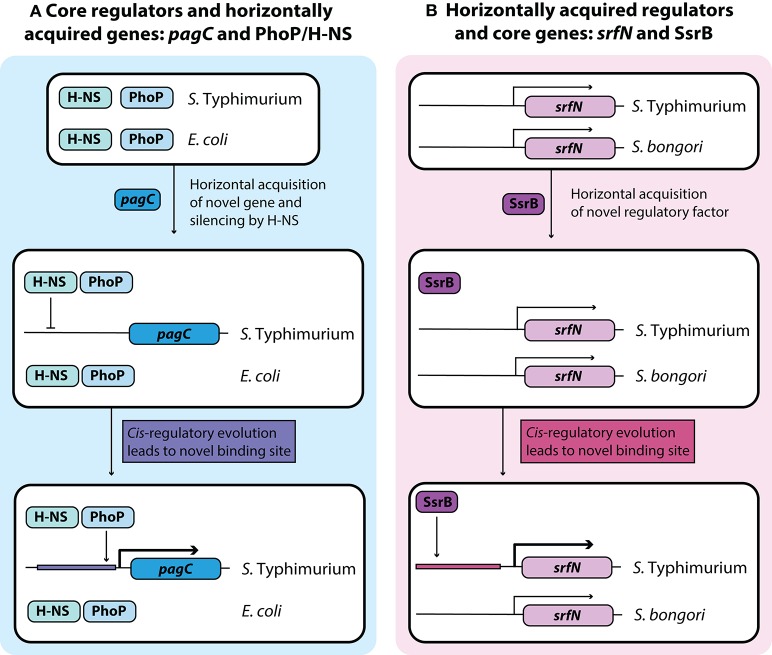
Development of novel regulatory pathways through transcriptional rewiring. **(A)** Horizontally acquired genes in *Salmonella* are silenced by H-NS, and *cis*-regulatory elements can undergo mutations to acquire binding sites that bring these genes under the control of a core virulence regulator such as PhoP. This counter-silencing allows for activation of virulence relevant horizontally acquired genes like *pagC* in infection relevant conditions. **(B)** Acquisition of the transcriptional regulator SsrB in *Salmonella* Typhimurium led to *cis*-regulatory evolution of core genes that allow them to come under the regulatory control of SsrB. This regulatory rewiring of core genes fine-tunes their expression under infection conditions with other SsrB regulated genes.

Horizontal gene transfer drives bacterial evolution by introducing large amounts of genetic variation in single events, giving rise to adaptation occurring in what many refer to as “quantum leaps” (Groisman and Ochman, 1996). However, in order for horizontally acquired genes to confer fitness advantages, their expression is subject to precise regulatory control such that they are deployed at the appropriate times to contribute beneficially to the organism. Here, we describe the regulatory circuitry within *Salmonella* that has evolved to integrate newly acquired genes into the existing flexible genetic networks that mediate pathogenesis (Figure 2).

### H-NS: the master silencer of horizontal transfer events

Although, the acquisition of genes through horizontal transfer has been essential to the evolution of *Salmonella* pathogenesis, the majority of foreign DNA is detrimental to bacteria (Buckling and Rainey, 2002; Navarre et al., 2007). Insertion within functional genes, overexpression of energetically taxing genes, and activation of unfavorable gene products are all examples of the drawbacks to horizontal gene transfer. Thus, the integration of these genes into core regulatory circuitry is critical for appropriate temporal gene expression and to ensure that these genes do not antagonize existing cellular functions. While this integration slowly proceeds via *cis*-regulatory mutation (discussed later), transcriptional silencing of horizontally acquired genes, termed xenogeneic silencing, protects the bacteria from these laterally acquired genes (Singh et al., 2016).

Seminal work over the past decade has identified H-NS, a DNA binding protein conserved across Gram-negative bacteria, as a broad repressor of horizontally acquired genes (Lucchini et al., 2006; Navarre et al., 2006). The mechanism of this repression relies on the intrinsic curvature of DNA rich in adenine and thymine (AT-rich; Navarre et al., 2006), which allows for H-NS binding in the minor groove, followed by nucleation and bridging along the DNA that ultimately blocks RNA polymerase (Ali et al., 2012). The existence of H-NS as a global sentinel of horizontally acquired genes has been speculated to contribute to the preferential retention of AT-rich horizontally acquired genes, as H-NS can mitigate the immediate harmful effects of lateral gene transfer (Dorman, 2007; Higashi et al., 2016).

Without compensatory mutations, deletion of *hns* tends to be lethal in *Salmonella* but not in closely related bacteria like *E. coli*, indicating the importance of H-NS to the biology and virulence of *Salmonella* (Navarre et al., 2006; Ali et al., 2014). Given the importance of horizontally acquired genes to the host-adapted lifestyle of *Salmonella*, research on H-NS has focused on the protein as a regulator of the pathogenic lifestyle. H-NS binds to SPIs 1-6 as well as other virulence-associated islets, blocking RNA polymerase from activating transcription from these loci (Stoebel et al., 2008). The importance of H-NS in pathoadaptation is further highlighted in *in vitro* evolution experiments, where mutations in *hns* are associated with loss of virulence determinants such as SPI-1 (Ali et al., 2014).

While H-NS is the most widely known silencer of virulence gene expression, it does not act alone upon horizontally acquired genes. Co-factors such as Hha and YdgT, and other nucleoid associated proteins like Fis contribute to the expression of horizontally acquired genes in *Salmonella*, including those that regulate virulence (Schechter et al., 2003; Silphaduang et al., 2007; Vivero et al., 2008). In contrast with core genes, which can be silenced by H-NS alone, the silencing of horizontally acquired genes such as those on the SPI loci or the pSLT plasmid additionally involve the co-factors Hha and YdgT (Vivero et al., 2008). Hha is specific to enteric bacteria and YdgT is specific to *E. coli* and *Salmonella*, yet are both involved in stabilizing H-NS at horizontally acquired promoters by mediating additional DNA contacts and allowing for H-NS aggregation (Coombes et al., 2005b; Ali et al., 2014). Similar to H-NS, the expression of Hha facilitates acquisition of foreign genes, thus contributing to pathogenic adaptation (Aznar et al., 2013). In summary, H-NS has been well-characterized as a buffer to help the bacterial cell tolerate new horizontal gene transfer events. Over time, these acquired genes have undergone further regulatory refinement for optimal expression in the host environment.

De-repression of H-NS by environmental stimuli is one way through which horizontally acquired genes can be activated in the right environments. Changes to DNA curvature induced by osmolarity or temperature shifts can modify the bridging activity of H-NS at specific promoters, allowing for transcription to proceed (Hinton et al., 1992). However, directed activation of virulence genes predominantly occurs through regulatory rewiring of H-NS binding elements to put these genes under the control of virulence regulators, or to allow for transcription factor binding that displaces H-NS bridges from the DNA. Examples of these methods have been identified for counter-silencing H-NS at horizontally acquired virulence genes in *Salmonella*, and their significance is discussed below.

### PhoP-PhoQ: assimilation of virulence determinants into a conserved system

Given the extent of xenogeneic silencing in *Salmonella*, the pathogenic lifestyle is highly reliant upon the evolution of regulators to counter-silence H-NS and relieve this repression (Stoebel et al., 2008). Many horizontally acquired genes become incorporated into the regulatory framework of two-component sensory systems, which are embedded in genetic networks to rapidly detect and respond to environmental cues (Wallis and Galyov, 2000). The PhoP-PhoQ regulatory system is highly conserved across bacterial species and governs several aspects of the *Salmonella* virulence program (Miller et al., 1989) with the regulation of ~5% of genes (Zwir et al., 2005; Harari et al., 2010). Consisting of a sensor kinase in the inner membrane (PhoQ), and response regulator in the cytoplasm (PhoP), this system senses low Mg^2+^ (García Véscovi et al., 1996), low pH (Bearson et al., 1997), and cationic antimicrobial peptides (Bader et al., 2005) within the stomach and small intestine to mediate rapid adaptation of *Salmonella* to the host environment. The detection of these environmental cues results in the positive regulation of PhoP-activated (*pag*) gene expression, and negative regulation of PhoP-repressed (*prg*) gene expression (Groisman, 2001). Additionally, PhoPQ activity is involved in the upregulation of both the PmrA-PmrB and SsrA-SsrB two-component systems, which are both important for intracellular survival (Gunn and Miller, 1996; Deiwick et al., 1999).

The majority of genes identified to be regulated by PhoP-PhoQ have been acquired by horizontal gene transfer (Groisman, 2001) and integrated into the PhoP regulon over evolutionary time. This suggests that horizontally acquired genes co-evolve with the *Salmonella* genome to become assimilated into existing core regulatory architecture, such that the spatiotemporal expression of virulence determinants is tightly controlled. Interestingly, PhoP appears to act differentially to regulate promoters acquired by horizontal gene transfer relative to those predicted to be ancestral (Will et al., 2014). A comparison of the promoter architectures between foreign and ancestral genes suggests that those that were horizontally acquired bind PhoP flexibly with high variability at a number of positions, whereas those that are part of the core genome interact with PhoP in a conserved manner at one binding site (Zwir et al., 2005, 2012). Furthermore, PhoP is capable of activating ancestral promoters directly via RNA polymerase holoenzyme interaction (Will et al., 2014), but horizontally acquired promoters only by counter-silencing of H-NS (Will et al., 2015). The importance of PhoPQ in regulating horizontally acquired genes is also evident from the divergence in PhoP targets despite the conservation of this regulatory system across diverse taxa. For example, *Yersinia pestis* (Grabenstein et al., 2006), *Shigella flexneri* (Moss et al., 2000), *Erwinia carotovora* (Flego et al., 2000), *Klebsiella pneumoniae* (Cheng et al., 2010), and *Sodalis glossinidius* (Toh et al., 2006) all contain PhoPQ and are severely attenuated for fitness in its absence, but have strikingly different lifestyles and highly distinct PhoP regulons (Groisman, 2001). These findings indicate that PhoPQ is a broadly conserved regulatory system that can flexibly integrate ancestral and acquired genes to accommodate bacterial lifestyles ranging from endosymbiosis to parasitism.

### SsrA-SsrB: *cis*-regulatory evolution within the dichotomous *Salmonella* species

The SsrA-SsrB two-component regulatory system activates the T3SS-2 and is essential for survival and replication within host immune cells (Fass and Groisman, 2009). SsrB can directly bind to genes within SPI-2, and outside of this island, and activate their transcription (Worley et al., 2000; Walthers et al., 2007). This activity is mediated by a flexible 18 base pair palindromic sequence in 7-4-7′ architecture upstream of SsrB-regulated genes, allowing SsrB to regulate ~5% of the Salmonella genome (Tomljenovic-Berube et al., 2010). Interestingly, in the absence of H-NS, SsrB is less required for SPI-2 gene expression, and relatively recent work has demonstrated that SsrB can directly displace H-NS polymers along DNA (Walthers et al., 2011). Additionally, SsrB mediates a key regulatory cross-talk between SPI-1 and SPI-2. For example, SPI-2-encoded SsrB downregulates SPI-1 genes by repressing HilA and HilD (Pérez-Morales et al., 2017), and reciprocally, SPI-1-encoded HilD upregulates SPI-2 gene expression by directly binding the ssrAB operon (Bustamante et al., 2008). This further demonstrates the cross-talk between pathogenicity islands in order to regulate the different lifestyles of *Salmonella* during infection.

The classical definition of *cis*-regulatory evolution rests upon the inevitable accumulation of mutations in non-coding DNA that drift in the nearly neutral range (Stone and Wray, 2001). Those mutations that generate a fitness-increasing quantitative output to alter gene expression may sweep to fixation, creating novel regulatory nodes that result in the flexible expansion of complex genetic networks (Wray, 2007). Adaptation within *cis*-regulatory elements is proposed to contribute to genetic tunability in response to environmental cues, a critical component of host colonization. However, up until recently, empirical evidence for this in the context of bacterial pathogenesis was largely lacking. Recent work has shown that mutations in non-coding DNA are targets for polymorphism-fixing selection to assimilate genes into the SsrB regulon, and that divergence in the regulatory patterns between *S. enterica* and *S. bongori* (which lacks SsrB) confers pathoadaptive fitness differences. For example, we demonstrated that following acquisition of a new regulatory system, the promoters that regulate ancestral genes can evolve responsiveness to the new transcription factor to fine-tune fitness in the host (Osborne et al., 2009). This involves rewiring the *cis*-regulatory element controlling the ancestral gene that generates phenotypic diversity among the bacterial population that is selective in the host setting. Regulatory evolution explains much of the organismal diversity among closely related animals in the context of developmental evolution (i.e., “evo-devo”) however these findings suggested that regulatory evolution might be a more broadly applicable evolutionary principle. Work by other groups has since verified and extended these findings, showing that regulatory evolution drives diverse bacterial traits including immune evasion (Tuinema et al., 2014), antibiotic resistance (Horii et al., 1999), and virulence (Li et al., 2009).

## The importance of gene loss to pathoadaptation in *Salmonella*

While the contribution of horizontally acquired genes in the pathoadaptation of *Salmonella* has been well-studied, the role of gene loss has received somewhat less attention. Bacterial adaptation to the host environment is understood to involve considerable genomic rearrangement and several studies have addressed the continuously fluctuating size of bacterial genomes, attributed largely to horizontal gene transfer, gene loss, and duplication (Bliven and Maurelli, 2012). We have described how *cis*-regulatory evolution mediates the assimilation of acquired virulence determinants into existing regulatory circuitry; gene loss is another process whereby certain genes may be inactivated to permit a pathogen's newly acquired virulence factors (Maurelli et al., 1998). Several studies have suggested that an intracellular lifestyle and host range specificity facilitate genomic degradation across the *Salmonella* serovars, resembling that which occurs in obligate intracellular endosymbionts that are entirely dependent on their eukaryotic hosts (Parkhill et al., 2001; Thomson et al., 2008; Nuccio and Bäumler, 2014). Within bacteria, gene loss proceeds by either genomic rearrangement or pseudogenization. We might predict that this occurs more readily within the host environment encountered by *Salmonella* during an infection, due to drastic population bottlenecks. These repeated reductions in population size force the random resampling of allelic variation, such that loss-of-function mutations accumulate more rapidly than expected (Nilsson et al., 2005; Albalat and Cañestro, 2016).

How is gene loss identified in bacterial genomes? Phylogenetic reconstruction is often sufficient to reveal the absence of genes when related species are compared to their last common ancestor. The presence of segregating variants with loss-of-function mutations in a population may also signify gene loss. However, these approaches are limited by the accuracy of phylogenetic inference, and additionally complicated in the case of prokaryotic species with genes lacking phylogenetic signal (Albalat and Cañestro, 2016). Exploring phylogenetic relationships with more evolutionary distance is a potential strategy to aid in the detection of reductive evolution. For example, the distantly related tsetse fly endosymbiont *S. glossinidius* possess a genomic region with strong sequence similarity to SPI-2 in *Salmonella*, and may be used to derive phylogenetic signal necessary for molecular evolutionary analyses. However, an alternative method to characterize patterns of gene loss is with experimental evolution studies that mimic the intracellular environment encountered by bacteria, to measure deletion rates and identify potential fitness advantages. Experiments of this nature are the largest contributors to our current understanding of the evolution of the salmonellae via gene loss.

Investigations of long-term experimental evolution have revealed high rates of deletion in bacteria conferring fitness advantages (Barrick et al., 2009; Khan et al., 2011). In experimentally evolved *Salmonella* populations simulating conditions of infection, genetic drift has been shown to drive RecA-independent gene deletions ranging from 1 to 202 kb over a strikingly brief period of time (Nilsson et al., 2005). Others have shown that selection is also capable of driving random deletions to fixation in the *Salmonella* chromosome that increase fitness as measured by growth rate (Koskiniemi et al., 2012). Furthermore, analyses of the more pathogenic Typhi and Paratyphi serovars have revealed low rates of purifying selection and recombination, but rather considerable loss-of-function gene mutations (Holt et al., 2008). A higher incidence of pseudogene formation and gene loss have been demonstrated in Paratyphi and Typhi relative to Typhimurium, perhaps due to their increased virulence and/or smaller host range (McClelland et al., 2004).

Why do gene loss mutations confer fitness increases in *Salmonella*? One hypothesis is that these readily occur in genes participating in pathways that become non-essential in new environmental conditions. As novel host adaptation occurs, neutral mutations may accumulate in these superfluous genes, mediating pseudogenization. Another proposed explanation for gene loss is that loss-of-function mutations occur exclusively in “antivirulence” genes: genes that are antagonistically pleiotropic and incompatible with horizontally acquired virulence determinants (Bliven and Maurelli, 2012). An example of this in *Salmonella* is the loss of the *lacI* repressor, which negatively regulates the lactose fermentation system in *E. coli*, a process that is not required by *Salmonella* residing in the gastrointestinal environment with access to dietary glucose (Eswarappa et al., 2009). The loss of this gene has been demonstrated to confer fitness advantages, as bacteria expressing it are capable of invasion but attenuated for survival within murine macrophages. It has been proposed that LacI was lost within *S*. Typhimurium to facilitate the acquisition of SPI-2, as it was found to repress virulence genes within this island and remains present in *S. bongori*, which lacks SPI-2 (Eswarappa et al., 2009). These findings suggest that LacI is an antivirulence gene that underwent selection for loss to promote an intracellular lifestyle.

## *Salmonella* infection biology in the post-genomics era

The “post-genomics” era has seen several advancements in next-generation sequencing to facilitate the rapid, cost-effective generation of completed microbial genome sequences. An increased emphasis on comparative genomics and transcriptomics of cells during infection has improved our ability to detect horizontal gene transfer events and identify the origins of virulence evolution (Thompson et al., 2006; Medini et al., 2008). The advent of RNA-sequencing (RNA-Seq) in particular has heralded an era of *Salmonella* research that has uncovered several novel aspects of innate immune evasion, as is evidenced by several recent transcriptomics experiments.

To identify the gene expression changes induced by host immune pressures, RNA-Seq was performed on *Salmonella* grown in 22 *in vitro* conditions approximating those presented by the immune system over the course of infection. This study identified upregulated expression in 86% of all *S*. Typhimurium genes (Kroger et al., 2013); subsequent work identified 31 genes that are differentially regulated between growth *in vitro* and within a murine macrophage model of infection (Srikumar et al., 2015). The regulatory nature of *Salmonella*-host interactions was characterized using RNA-Seq to explore the targets of 18 key transcriptional regulators within *S*. Typhimurium, which identified 1,257 genes with altered expression profiles (Colgan et al., 2016). Interestingly, single-cell RNA-Seq within macrophages identified that *Salmonella* undergoing active replication shifts macrophage metabolism from an M1 to an M2 polarization state, perhaps capitalizing upon the anti-inflammatory environment in M2 macrophages (Saliba et al., 2016). These *Salmonella*-based RNA-Seq experiments have all explored gene expression profiles during intracellular survival and upon exposure to components of host immunity, as well as helped to identify the genome-wide targets of core transcription factors and how they link to horizontally acquired genes.

## Summary

The interaction of *Salmonella* with the host during an infection requires sensing the host environment, activating genes in response to host antimicrobial defenses, and modifying the host to make it more amenable for colonization. There are a number of core genes that contribute to pathogenesis, such as motility genes required for chemotaxis within the gut lumen, fimbrial adhesins involved in mediating host cell contact, and metabolic genes that allow for nutrient acquisition in the competitive anaerobic gut environment. However, the biology of *Salmonella* in the context of infection is strongly driven by the acquisition of genes through horizontal gene transfer. The identification of SPI-1, shared by all *Salmonella* species and subspecies, as critical for bacterial mediated endocytosis into epithelial cells initiated decades of research into the role of horizontally acquired genes in *Salmonella* infections. A combination of tissue culture infections, genetic manipulation of *Salmonella*, and *in vivo* infection models have been the mainstay of research approaches to broaden our understanding of how SPIs contribute to *Salmonella* infection biology. This review highlighted the central contributions of the most recently identified pathogenicity islands in *S*. Typhimurium, and tied these findings into the overarching cellular biology of a *S*. Typhimurium infection. Our summaries of these virulence determinants are not comprehensive, as their precise molecular mechanisms have yet to be fully characterized. Although a number of the SPIs and phage associated genes have been identified as playing a role in virulence or survival within host cells, much remains to be understood in how these interact with other *Salmonella* genes and with host processes.

We have additionally discussed the complex interplay between horizontal gene transfer and *cis*-regulatory evolution, and described the role of these two biological processes in the promotion of patho-adaptive change. Over evolutionary time, the virulence program of *Salmonella* has been shaped by the acquisition of pathogenicity islands and phage-associated genes, furthering its divergence from to its closest relative, *E. coli*, and allowing for novel mechanisms of host invasion and resistance. Concurrently, considerable *cis*-regulatory change has occurred in the *Salmonella* genome to integrate horizontally acquired genes and ancestral core genes into new regulatory circuitry, to control their expression such that fitness is optimized. Evolutionary biologists have long appreciated the importance of *cis*-regulatory evolution in the generation of genetic variation to ultimately drive adaptive change; we have elaborated this to emphasize the impact of this process in increasing bacterial pathogenicity. The assimilation of newly acquired genes into the regulons of H-NS, PhoP-PhoQ, and SsrA-SsrB, has allowed for the expansion of flexible genetic networks that mediate bacterial pathogenesis and confer pathoadaptive fitness differences. We have also described the genomic degradation and gene loss that occurs alongside the horizontal acquisition of novel genes, due to a combination of relaxed selection and antagonistic pleiotropy. These findings highlight the dynamic genome that underpins the evolution of bacterial pathogenesis. In the age of antibiotic resistance when even the very process of evolution itself is a potential new target (Smith and Romesberg, 2007; Zaneveld et al., 2008; Rasko and Sperandio, 2010), understanding these processes in depth and with quantitative measures will become increasingly important.

## Author contributions

All authors listed have made a substantial, direct, and intellectual contribution to the work, and approved it for publication.

### Conflict of interest statement

The authors declare that the research was conducted in the absence of any commercial or financial relationships that could be construed as a potential conflict of interest.
